# Laser-Assisted Synthesis of Composite Nanoparticles of Perovskite BaTiO_3_ in Aqueous Solutions and Their Optical Properties

**DOI:** 10.3390/ma13184086

**Published:** 2020-09-14

**Authors:** Ekaterina V. Barmina, Bulat A. Mukhametyanov, Oleg V. Uvarov, Igor I. Vlasov, Oleg S. Kudryavtsev, Yurii L. Kalachev, Evangelos Skoulas, George Kourmoulakis, Valeriy V. Voronov, Emmanuel Stratakis, Georgy A. Shafeev

**Affiliations:** 1 Prokhorov General Physics Institute of the Russian Academy of Sciences, 38, Vavilov street, 119991 Moscow, Russia; barminaev@gmail.com (E.V.B.); mukhametyanov2010@yandex.ru (B.A.M.); uvarov@kapella.gpi.ru (O.V.U.); vlasov@kapella.gpi.ru (I.I.V.); leolegk@mail.ru (O.S.K.); kalachev@kapella.gpi.ru (Y.L.K.); voronov@lst.gpi.ru (V.V.V.); 2 Ιnstitute of Electronic Structure and Laser, Foundation for Research and Technology-Hellas, Heraklion, 71110 Crete, Greece; skoulasv@iesl.forth.gr (E.S.); geokourm@iesl.forth.gr (G.K.); stratak@iesl.forth.gr (E.S.); 3 Department of Materials Science and Technology, University of Crete, Heraklion, 70013 Crete, Greece; 4 Department of Physics, University of Crete, Heraklion, 70013 Crete, Greece; 5 National Research Nuclear University MEPhI (Moscow Engineering Physics Institute), 31, Kashira Hwy, 115409 Moscow, Russia

**Keywords:** laser ablation, liquid, nanoparticles, perovskite, BaTiO_3_, composite nanoparticles

## Abstract

Experimental results are presented on laser-assisted synthesis of composite nanoparticles of perovskite BaTiO_3_ with gold nanoparticles using the technique of laser ablation in water and aqueous solution of hydrogen peroxide. Nanoparticles of BaTiO_3_ are generated by near IR laser radiation with pulse durations of 170 fs, 1 ps, and 200 ns. Nanoparticles of barium titanate BaTiO_3_ (BTO) have tetragonal structure for all used pulse durations. Two ways of synthesis are tested. In the first one a gold target is ablated in the colloidal solution of BaTiO_3_ nanoparticles. The second way consists of laser exposure of the mixture of colloidal solutions of nanoparticles of BaTiO_3_ and Au. Synthesized composite nanoparticles are characterized by optical spectroscopy, Raman spectroscopy, X-Ray diffractometry, and Transmission Electron Microscopy. Composite BaTiO_3_-Au nanoparticles have the absorption band in the visible range of spectrum and demonstrate plasmonic luminescence.

## 1. Introduction

Hydrogen is an attractive alternative to hydrocarbon fuels due to its low environmental impact. One of the possible ways to produce hydrogen is photocatalytic water splitting under sunlight irradiation. Many materials had been tested for this approach, among them oxides [1,2].

Perovskites are promising materials for applications in photocatalytic water splitting and solar cells [3,4,5]. Perovskites with nanosized Au nanoparticles are efficient for hydrogen production [6].

Hybrid organic perovskites can be described by the formula ABX_3_, in which A is an organic cation, B is a metal cation (such as Pb), and X is a halogen anion (such as Cl, Br, or I) [7]. Some halogen ions, such as Br or I, lead to absorption of these hybrid perovskites in the visible range of spectrum. This makes them attractive for application in solar cells [8].

Applications of perovskites require development of methods of their dispersion with preservation of their crystallinity and optical properties. Chemical synthesis of perovskites, including organic perovskites, provides powders with high dispersion but frequently corresponding nanoparticles are amorphous.

Laser ablation of bulk perovskites in liquid and further laser fragmentation of generated powders is a well-known physical technique. This approach can be used for preparation of nanoparticles of large variety of metals. Laser ablation in liquids is characterized by high temperatures and transient pressures. In case of perovskites with their multi-element composition this may lead to the deviation of the crystallinity and stoichiometry of generated nanoparticles from that of the initial target. Laser fragmentation of micro-powders of organic perovskites in *n*-hexane was successfully realized in [9]. Photoluminescent properties of the generated nanoparticles (NPs) of organic perovskites correspond to properties of the starting material. Disadvantage of halogen-containing perovskites is their weak thermal stability, namely, halogen atom leaves the compound even under moderate temperature.

There is a wide class of inorganic perovskites. For example, Barium Tantalum Oxynitride BaTaO_2_N is an attractive material for solar splitting of water since this compound has absorption band in the visible range of spectrum [10]. In this work this perovskite was synthesized via solid-state reaction at high temperature and then was subjected to laser fragmentation in liquid jet. It was found that under several cycles of laser fragmentation the obtained nanoparticles have non-stoichiometric composition such as BaTaO_x_N_y_ due to partial loss of oxygen and nitrogen during laser fragmentation. However, the perovskite powder generated in this way does show the photo-electrochemical activity.

Barium titanate BaTiO_3_ (BTO) is the most common perovskite ferroelectric materials, which is used as capacitor, ferroelectric memory and so on because of its excellent dielectric, piezoelectric and ferroelectric properties [11]. Additionally, nanoparticles of BTO can be used for drug delivery due to its high biocompatibility [12]. Optical properties of BaTiO_3_ and other perovskite (SrTiO_3_) have been explored in detail and are well-known [13]. BTO is a wide bandgap compound (bandgap of 3.2 eV), and it has no absorption in the visible range of spectrum. Barium titanate (BTO) films can be prepared by sol-gel spin-coating technique [14]. The results indicate that the BTO films have tetragonal symmetry. The bandgap of synthesized BTO films depends on the post-annealing temperature. To introduce the absorptivity of BTO the chemical synthesis of BTO nanoparticles with Au nanoparticles was achieved via sol-gel process at ambient temperature. Au NPs are embedded in the amorphous BaTiO_3_ matrix. Tentative energy band diagram of Au NPs on BTO nanoparticles is reported in [15]. However, low temperature prevents the formation of reliable contact of Au nanoparticles with nanoparticles of BaTiO_3_ (BTO). Nanocomposites of BTO-Au prepared by chemical route show excellent activity in immunity tests [16].

Laser ablation in liquids is an alternative technique for fabrication of various nanoparticles and nanocomposites. This physical method has been applied for generations of large diversity of nanoparticles, mostly of noble metals [17,18,19] but also for nanoparticles of more chemically active metals, such as Al and Ti [20,21,22,23] in non-aqueous liquids. Duration of laser pulses that are used in the technique of laser ablation in liquids spans from femtoseconds to microseconds. Comprehensive review of recent developments of the method of laser ablation in liquids has been done in recent publication [24].

In this work, we present our results on laser-assisted synthesis of nanocomposites of nanoparticles of inorganic perovskite BTO with Au. Laser synthesis of BTO-Au nanocomposite solves two problems. First, Au nanoparticles are characterized by absorption band in the visible due to plasmon resonance. Second, contact of Au with BTO provides Schottky barrier that separate the light-generated charge carriers.

Preliminary results on laser synthesis of BTO-Au nanocomposites have been published in our recent paper [25]. The extended version of the paper is presented here. The aim of the work is laser-assisted synthesis of perovskite BTO nanoparticles that possess permanent temperature-stable absorption in the visible range of spectrum corresponding to the Au NPs.

## 2. Experimental Setup

Two approaches were used for the synthesis of BTO-Au nanocomposites:

1. Laser ablation of a bulk Au target in aqueous colloidal solution of BTO NPs. In this case the concentration of BTO remains constant while the concentration of Au NPs increases with the increase of ablation time, and the content of Au NPs increases with the ablation time.
2. Laser exposure of the mixture of colloidal solutions of Au and BTO nanoparticles prepared separately. In the latter case the laser exposure of the mixture of individual colloids was performed by the scanning laser beam focused into the liquid from the bottom through a glass window transparent for laser radiation. The synthesis of the composite is accompanied by simultaneous laser fragmentation of both BTO and Au NPs.

Two types of laser sources were used. The first one was Yb: KGW laser (Pharos, Vilnius, Lithuania) operating either at 170 fs pulse duration or 1 ps pulse duration at laser wavelength of 1030 nm and repetition rate of 1 kHz. The focal spot was 30 μm and the laser fluence on the target was of 14 J/cm^2^. In this case the BTO pellet placed into a vessel filled with working liquid was displaced under the laser beam focused on pellet surface with velocity of 1 mm/s. The second laser source was a fiber Yb laser (Ateko, Moscow, Russia) operating at 1060–1070 nm wavelength range and with pulse duration of 200 ns. The laser beam was scanned across the target surface at a speed of 100 mm/s using a galvo-mirror system and F-Theta objective (F = 204 mm). Geometry of laser scanning was a rectangle 5 × 8 mm^2^. The laser beam was scanned across the target surface at a speed of 100 mm/s using a galvo-mirror system. The colloidal solutions of individual nanoparticles of both Au and BTO were generated using this laser source at this step.

Nanoparticles of BaTiO_3_ (NPs of BTO) were generated using the well-known technique of laser ablation in liquids [17,18,19,20,21,22,23]. Sintered pellet made of BaTiO_3_ powder with some residual traces of BaCO_3_ and TiO_2_ was used as the ablation target. It was expected that BTO may lose some part of oxygen being dispersed as NPs of BTO, and the laser ablation was performed in two liquids: in H_2_O (MQ grade) and in 30% H_2_O_2_ in H_2_O. The choice of H_2_O_2_ was stipulated by the suggestion that BaTiO_3_ may lose some oxygen upon laser ablation [26,27]. H_2_O_2_ was the agent that may provide additional oxygen around the laser spot on the target under liquid.

The morphology of NPs was studied with the help of Transmission Electron Microscope Carl Zeiss (Carl Zeiss, Jena, Germany). Raman spectra of evaporated on Si substrate NPs were acquired using a Raman spectrometer (GPI RAS, Moscow, Russia) with excitation wavelength of 473 nm. The same spectrometer was used for acquiring luminescence spectra of the samples. X-ray diffraction patterns were recorded using an X-ray diffractometer Bruker D8 Discover A25 DaVinci Design (Bruker, Rome, Italy). Reflection spectra of generated NPs evaporated on Al_2_O_3_ ceramics were acquired with the help of Shimadzu-3600 spectrometer (Shimadzu, Tokyo, Japan) with respect to reflectivity of Al_2_O_3_ ceramics in the 200–700 nm spectral range.

## 3. Results

### 3.1. Generation of BTO Nanoparticles

BaTiO_3_ is a non-linear optical material, and one can observe green radiation from the BTO pellet when it is outside of the focal plane of laser beam. This radiation is not visible anymore when the laser beam is tightly focused on the target. In this case, only bright plasma on the target is visible.

**Figure 1:**
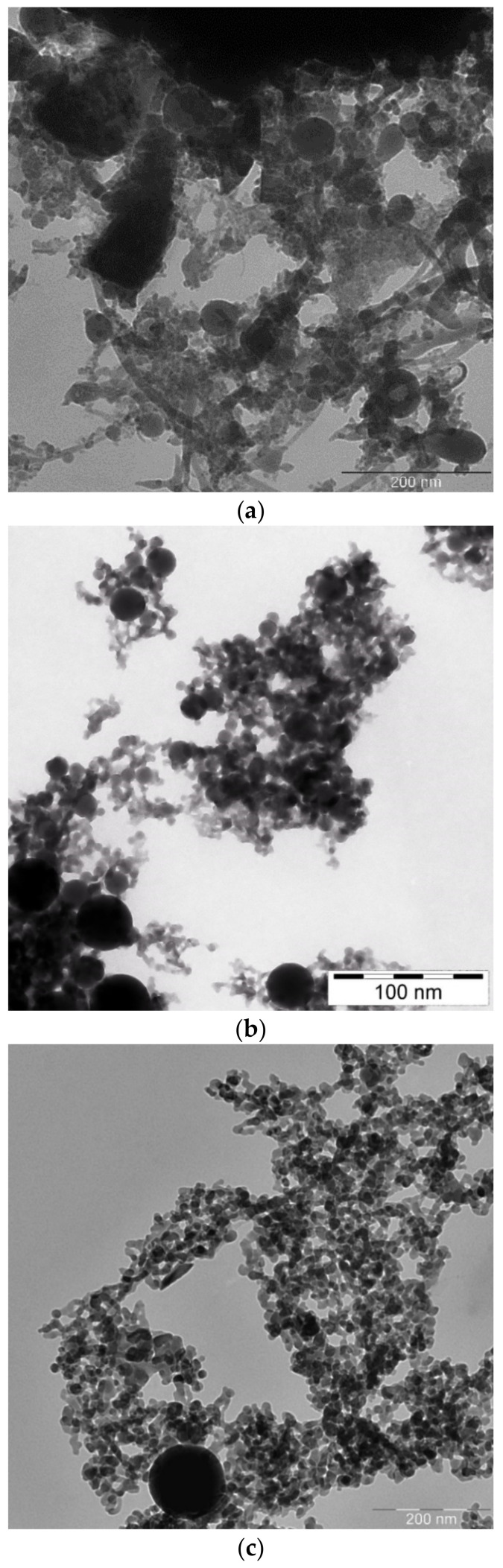
TEM view of nanoparticles (NPs) of barium titanate BaTiO_3_ (BTO) generated by ablation in H_2_O at pulse duration of 1 ps (**a**), in H_2_O at pulse duration of 200 ns (**b**), and ablation in H_2_O_2_ at pulse duration of 200 ns (**c**). Scale bars denote 200 (**a**,**c**) and 100 nm (**b**).

Transmission Electron Microscope (TEM) images of laser-generated BTO nanoparticles generated at various pulse durations and different liquids are shown in Figure 1.

One can see that well-defined and spherically shaped nanoparticles of BTO are generated only using nanosecond laser pulses. BTO NPs generated with 1 picosecond pulses are characterized with stochastic shape that may be due to highly non-equilibrium process of laser ablation that proceeds rather via mechanical damage of the target than its melting. On the contrary, BTO NPs generated by 200 ns laser pulses have a spherical shape. This means that generation of BTO NPs in this case proceeds through melting of the target. Therefore, in the laser synthesis of BTO-Au composites only BTO NPs produced by ablation with 200 ns laser pulses were used.

**Figure 2:**
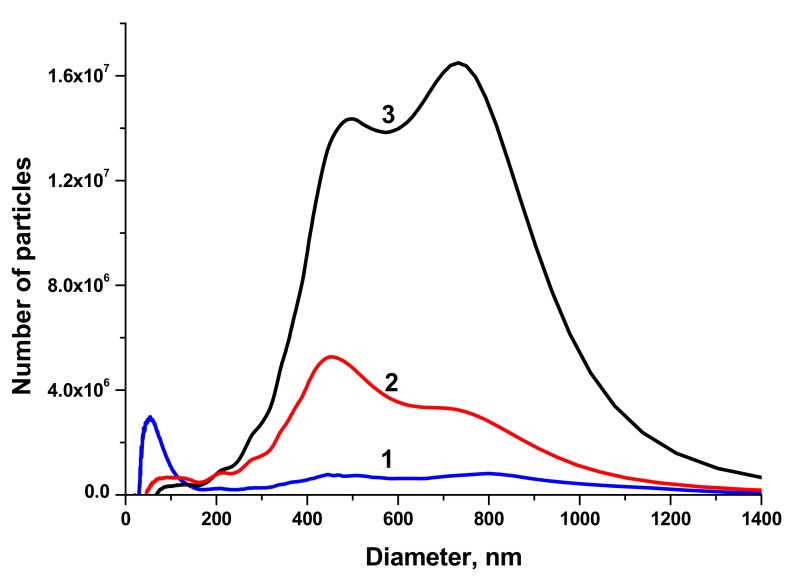
Size distribution of BaTiO_3_ NPs generated by laser ablation in H_2_O with pulse duration 200 ns (1), in H_2_O with pulse duration 1 ps (2), in H_2_O_2_ with pulse duration 1 ps (3). The volume of the colloidal solution injected into the centrifuge is of 50 μL.

The size distributions of BTO NPs generated by laser ablation in different liquids and various pulse durations are shown in Figure 2.

NPs generated with 1 ps laser pulses have two maxima of the size distribution around 450 and 800 nm, the fraction of small NPs is negligible. This can be attributed to the generation of relatively large fragments from the BTO pellet that was sintered from micro-powder of BTO. This agrees with the suggestion made above about mechanical damage of the target under action of short laser pulses. On the contrary, BTO NPs generated with 200 ns pulses have substantial fraction of NPs with size around 50–60 nm. The concentration of such NPs in the solution is of order of 10^11^ cm^−3^.

**Figure 3:**
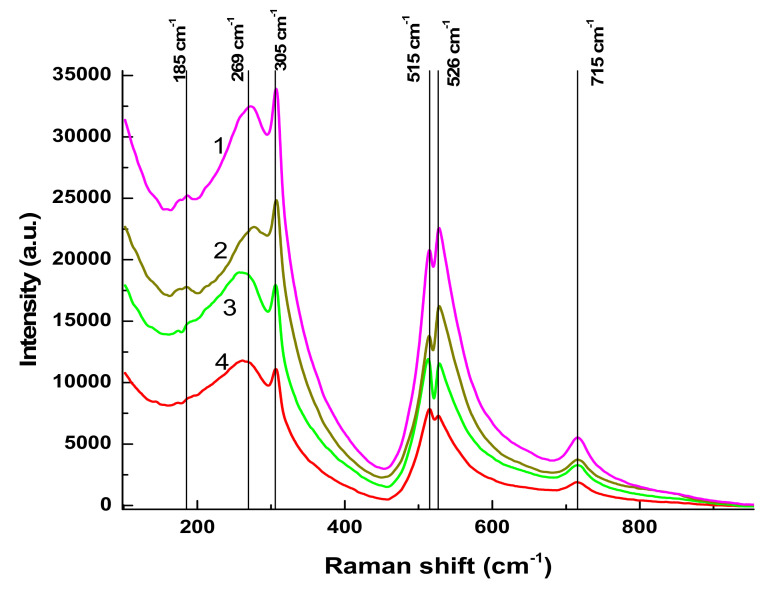
Raman spectra of BTO NPs obtained at various pulse durations. (1)—BaTiO_3_ in H_2_O, 1 ps; (2)—BaTiO_3_ in H_2_O_2_, 1 ps; (3)—BaTiO_3_, ablation time of 45 min at 170 fs in H_2_O; (4)—BaTiO_3_ ablation time of 45 min at 170 fs in H_2_O_2_. Excitation wavelength is of 473 nm.

Raman spectra of BTO NPs generated by laser ablation with short (170 fs and 1 ps) laser pulses and then evaporated on a Si substrate are presented in Figure 3. The observed Raman modes at 305 cm^−1^ and 715 cm^−1^ agree with the literature and are characteristic of the tetragonal phase of BTO. A similar spectrum is observed with BTO NPs ablated with pulse duration of 200 ns (see Section 3.3 below).

**Figure 4:**
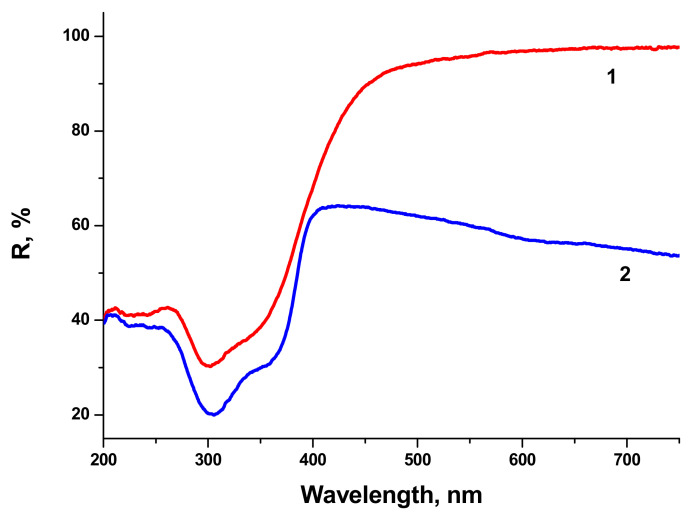
Reflectivity spectra of BTO NPs evaporated on alumina plate with respect to alumina ceramics. Ablation in H_2_O_2_ (1), ablation in H_2_O (2). Yb fiber laser, pulse duration of 200 ns.

There is minor difference in Raman spectra between BTO NPs generated with 170 fs and 1 ps pulses in the vicinity of 180 cm^−1^. Otherwise the spectra are similar both in case of laser ablation in H_2_O and H_2_O_2_. Reflectivity spectra (mirror reflection) of BTO NPs evaporated on alumina plate are shown in Figure 4. The difference in reflectivity of NPs ablated in H_2_O and H_2_O_2_ is clearly visible. NPs obtained by ablation in H_2_O have lower reflectivity both in UV and visible range of spectrum compared to NPs generated by ablation in H_2_O_2_. Reflectivity is the only one analytical technique by which this difference is observed in this work. Additional absorption is associated with the loss of oxygen from BaTiO_3_ at elevated temperature during laser ablation [26,27]. A sharp decrease of reflectivity below 400 nm corresponds to inter-band absorption in BaTiO_3_.

Very similar reflectivity spectra are also observed for the BTO pellet itself, laser-ablated surface in H_2_O looks gray due to loss of oxygen (not shown here).

**Figure 5:**
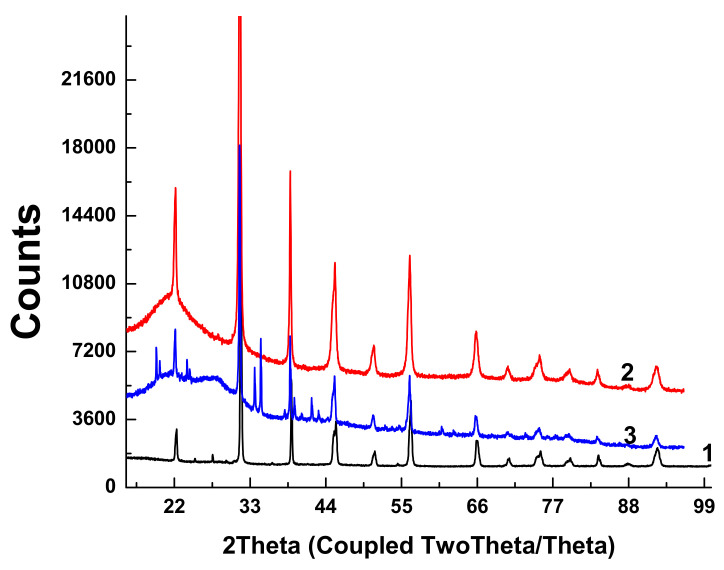
X-ray diffractograms of the initial target pellet (1), BTO NPs generated by ablation in H_2_O with 1 ps pulses (2), and BTO NPs generated by ablation in H_2_O with 200 ns pulses (3). Additional diffraction peaks in the range 20–30° are due to the substrate.

X-ray diffractograms of the initial BTO pellet and that of evaporated BTO NPs generated with laser pulses of various durations are presented in Figure 5.

Diffractograms of some samples of BTO NPs contain peaks of BaCO_3_. This is probably due to the inhomogeneity of the initial pellet, since BaTiO_3_ is synthesized by joint sintering of BaCO_3_ and TiO_2_ at a high temperature.

Diffractograms for BTO NPs generated by ablation in H_2_O_2_ with laser pulse duration of either 1 ps or 200 ns are identical (not shown here). It is interesting to note that colloidal solution of BTO NPs does not convert IR laser radiation to the second harmonics unlike the initial BTO pellet. This is probably due to the small size of NPs insufficient for frequency doubling. The colloidal solutions of BTO NPs prepared in this way were further used for synthesis of BTO-Au nanocomposites.

### 3.2. Generation of Au Nanoparticles

**Figure 6:**
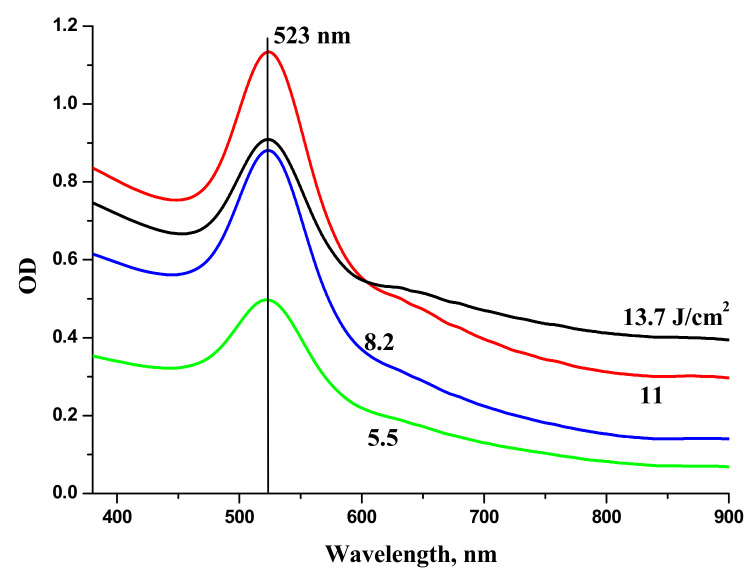
Extinction spectra of Au NPs generated by ablation of Au target with pulse duration 200 ns in H_2_O with various fluences on the target surface. Laser fluence on the target is indicated at each spectrum. Copied from reference [25] with copyright permission from Journal of Physics of Wave Phenomena Springer publisher.

Au NPs were generated by laser ablation of a bulk Au target in H_2_O. The series of extinction spectra (optical density) of Au NPs in H_2_O generated at various laser fluences on the target is shown in Figure 6. One can see that the extinction coefficient of the solutions in red region increases with the increase of laser fluence. Red “wing” in the extinction spectrum of Au colloids corresponds to generation of elongated Au NPs. At lower fluence, only one peak around 520 nm is observed that corresponds to transvers plasmon resonance of spherical Au NPs [28].

The increase of optical density of Au NPs in the range 600–800 nm is associated with elongated Au NPs and corresponds to the overlaps of longitudinal plasmon resonances of elongated Au NPs with various aspect ratios [29]. Au NPs used for laser synthesis of BTO-Au nanocomposite were generated at moderate laser fluence on the target in order to provide mostly spherical Au NPs. Extinction spectra of NPs generated by laser ablation of Au in H_2_O_2_ virtually coincide with those for Au ablated in H_2_O.

**Figure 7:**
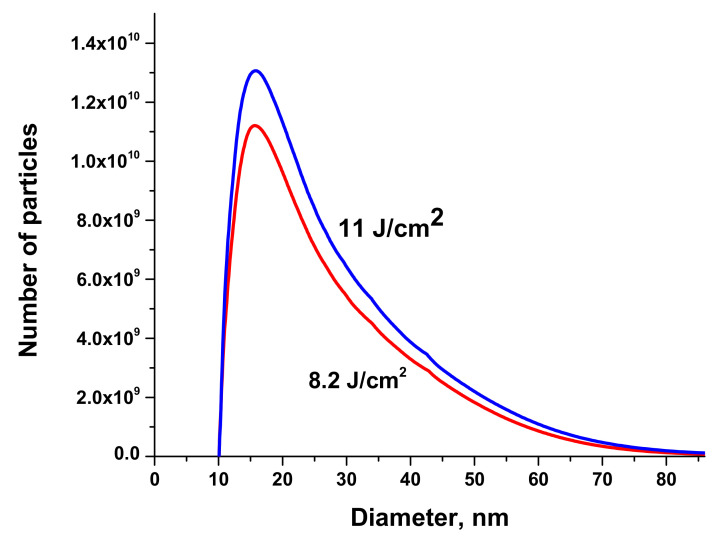
Size distribution of Au NPs generated by ablation of a bulk Au target in H_2_O at various fluences on the surface of target. Laser fluence is indicated at each curve. Size distribution was determined using disk measuring centrifuge. Copied from reference [25] with copyright permission from Journal of Physics of Wave Phenomena Springer publisher. One can see that most of Au NPs have the diameter between 15 and 20 nm. Their colloidal solution in H_2_O was used in further laser synthesis of nanocomposites BTO-Au.

Size distributions of Au NPs are presented in Figure 7. The volume of the solution injected into the centrifuge was 50 μL.

### 3.3. Laser Synthesis of BTO-Au Nanocomposites

**Figure 8:**
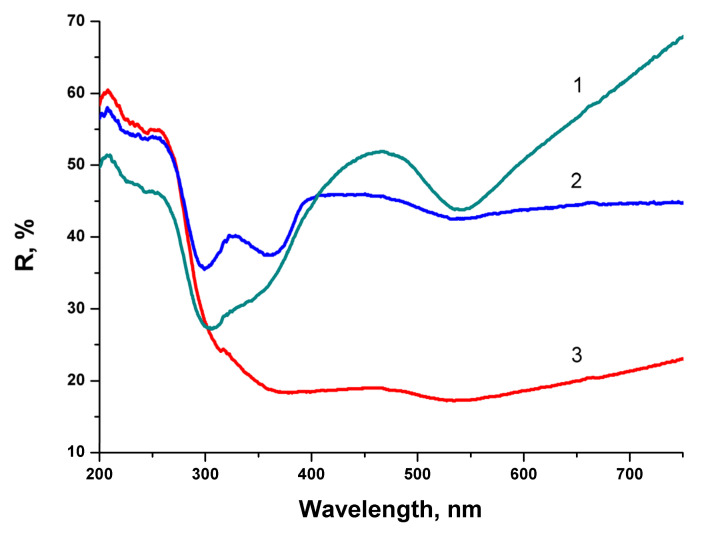
Reflectivity spectra of BTO-Au NPs with respect to alumina ceramics generated by laser ablation of Au target in aqueous solution of BTO NPs for: 6 min (1), 10 min (2), 15 min (3). Copied from reference [25] with copyright permission from Journal of Physics of Wave Phenomena Springer publisher.

Laser ablation of a bulk Au target in the aqueous colloidal solution of BTO NPs leads to formation of composite NPs. The appearance of composite NPs depends on the time of laser ablation. The relative content of Au increases with ablation time almost linearly. The series of reflectivity spectra of composite BTO-Au NPs is shown in Figure 8.

At relatively short time of laser ablation (6 min) the reflectivity spectrum is characterized by two minima. The first one near 300 nm corresponds to pure BTO NPs (see Figure 4). The second is at 540 nm and corresponds to plasmon resonance of Au NPs (see Figure 6). The shift in the peak position from 523 nm to 540 nm is due to the fact the Au is situated on BTO NPs. With the increase of ablation time both minima are still visible but are less pronounced. No minimum in reflectivity that corresponds to BTO NPs is not visible anymore at ablation time of 15 min. This indicates complete screening of BTO surface by Au NPs. Low reflectivity in this sample is due to destructive interference of the light in Au NPs.

**Figure 9:**
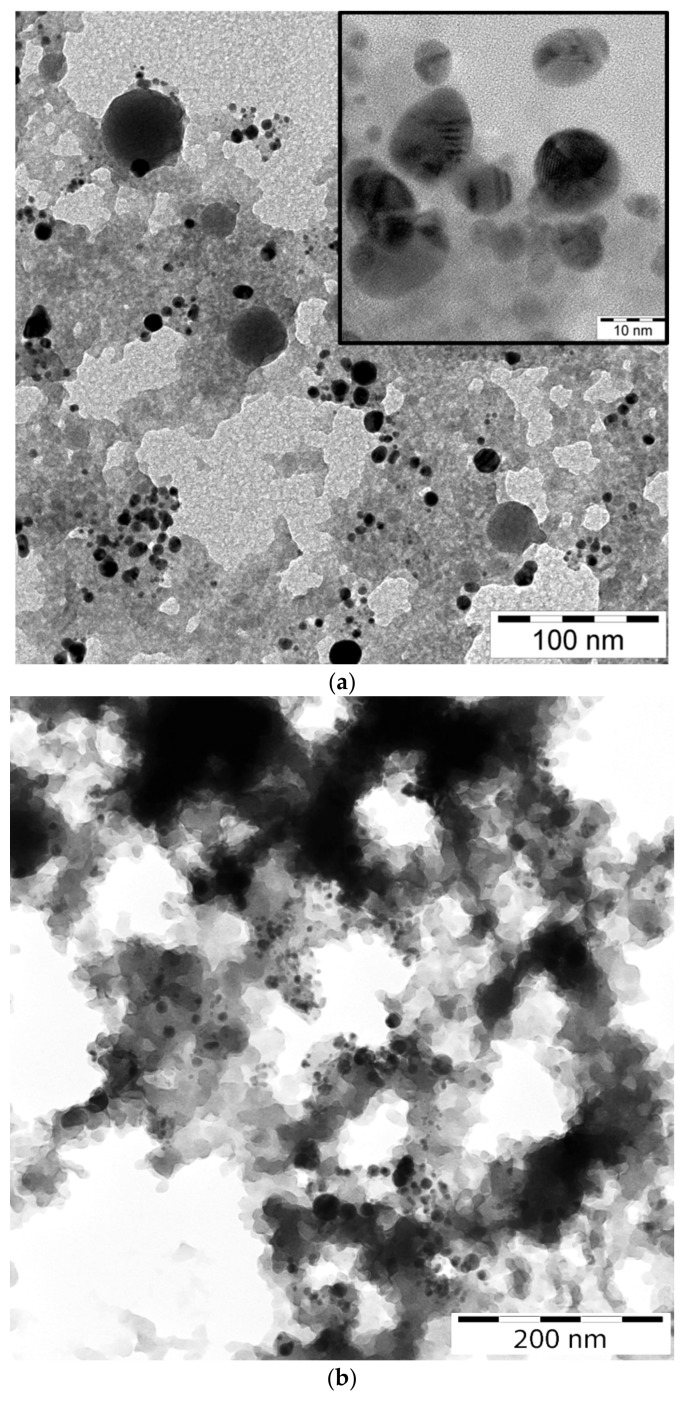
TEM images of BTO Au NPs. Laser ablation of Au target in the colloidal solution of BTO NPs with enlarged view in the inset (**a**) Laser ablation time is of 6 min. Laser exposure of the mixture of colloidal solutions of Au and BTO in H_2_O (**b**). Scale bar denotes 100 and 10 nm in the inset (**а**) and 200 nm (**b**).

TEM images of nanocomposite NPs BTO-Au synthesized by the two approaches are presented in Figure 9. Au NPs are visible due to their higher contrast compared to BTO NPs. Au NPs are on average 10 nm in diameter in case of ablation of Au target in BTO NPs colloidal solution. Relatively large spherical NPs of BTO are also visible.

**Figure 10:**
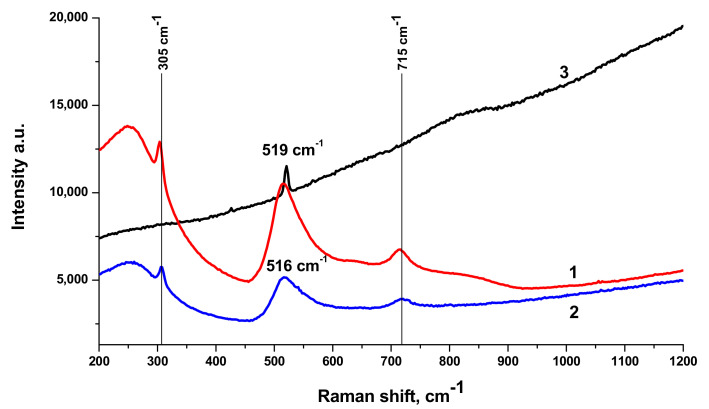
Raman spectra of: BTO NPs generated by laser ablation in H_2_O for 15 min (1), Au-BTO NPs generated by laser ablation of Au target in aqueous solution of BTO NPs for 10 min (corresponds to previous picture) (2), Au-BTO NPs generated by laser ablation of Au target in aqueous solution of BTO NPs for 15 min (corresponds to Figure 9) (3). Copied from reference [25] with copyright permission from Journal of Physics of Wave Phenomena Springer publisher.

Raman spectra of BTO-Au nanocomposite along with Raman spectrum of pure BTO NPs generated with 200 ns laser pulses are shown in Figure 10. Spectrum of pure BTO NPs virtually coincides with that generated with short laser pulses (see Figure 3). One can also conclude that presence of Au NPs supported on BTO NPs does not alter the Raman spectrum in observable way. With the increase of laser ablation time that is at higher Au content in the nanocomposite only the strongest peak at 516 cm^−1^ is observed and is upshifted to 519 cm^−1^. At the same time, Raman signal is characterized by gradual increase with the increase of wavenumber. This is typical of photoluminescence of the composite at high Au content. This feature is not observed in pure BTO NPs.

**Figure 11:**
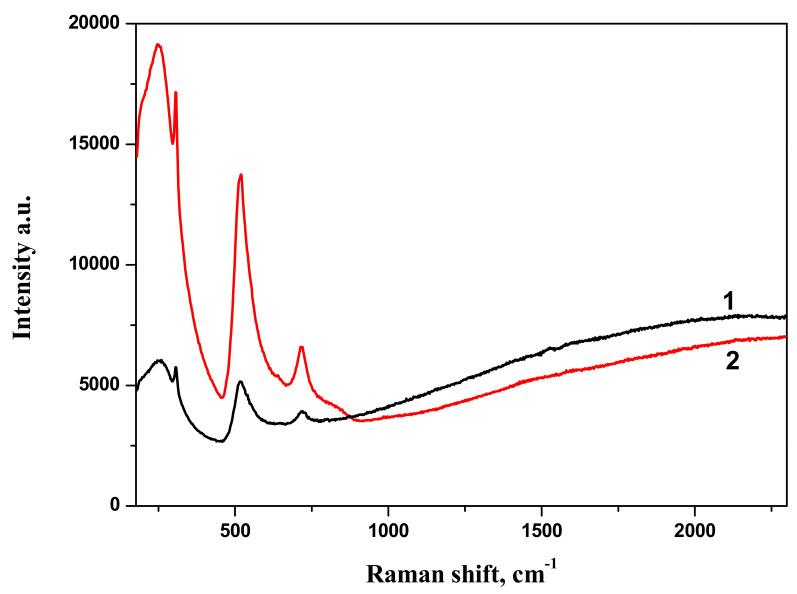
Raman spectra of composite nanoparticles BTO-Au obtained by ablation of Au target in the aqueous colloidal solution of BaTiO_3_ nanoparticles (1), irradiation of the mixture of aqueous colloidal solutions of Au and BTO NPs (2). Copied from reference [25] with copyright permission from Journal of Physics of Wave Phenomena Springer publisher. Luminescence spectra of NPs of pure BTO and BTO-Au nanocomposites with various Au content are presented in Figure 12.

The comparison of Raman spectra of BTO-Au nanocomposites synthesized by both approaches is presented in Figure 11. Both spectra are characterized by similar spectral features.

Luminescence spectrum of pure BTO NPs is very wide, which corresponds to wide absorption band of BTO NPs ablated in H_2_O. Luminescence spectra of BTO-Au nanocomposites have higher intensity and are characterized by maximum at 540 nm. This corresponds to plasmonic luminescence of nanosized Au [30].

**Figure 13:**
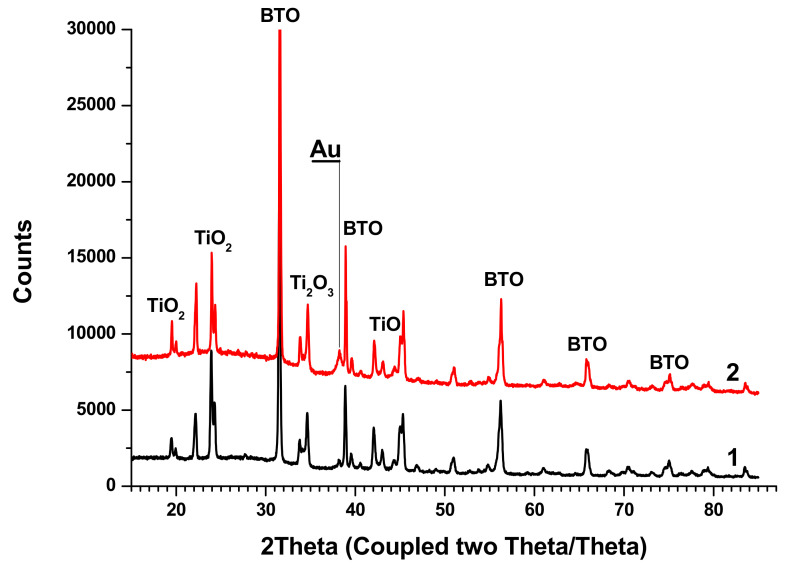
X-ray diffractograms of the composite Au-BTO NPs obtained by: laser ablation of Au target in colloidal solution of BTO NPs with 200 ns pulses (1), irradiation of the mixture of colloids of Au and BTO NPs with 200 ns pulses (2).

XRD analysis of BTO-Au nanocomposites synthesized by both approaches is shown in Figure 13.

The peaks of BTO are clearly visible, and their position is the same as in the diffractograms of pure BTO (Figure 5). Relatively small Au peaks can be distinguished. Additionally, peaks of several Ti oxides are presented.

## 4. Discussion

Laser ablation of BTO in H_2_O and H_2_O_2_ leads to the formation of NPs of BTO. Raman and XRD analysis (see Figure 3 and Figure 5) confirm that these NPs are in stable tetragonal crystallographic modification. Same modification is realized independently with various laser pulse durations, either of 1 ps or 200 ns (Figure 5). Only one analytical technique shows the difference between laser ablation of BTO pellet in either H_2_O or H_2_O_2_, namely, the reflectivity spectra. Additional absorption of BTO ablated in H_2_O is related to the loss of oxygen during laser ablation. The loss also may take place even under laser ablation of some oxides in air [26]. This was demonstrated for several oxides, including lead zirconate titanate (PZT). Laser ablation of solids in aqueous solutions is accompanied by generation of molecular H_2_ and O_2_ [24,31]. Their formation is due to dissociation of H_2_O molecules by the impact of electrons from laser-produced plasma. The equilibrium is shifted towards H_2_, and the whole atmosphere is oxygen-deficient.

Raman spectra of BTO-Au obtained by two methods are virtually identical and Au NPs do not alter the crystallinity of BTO (Figure 5 and Figure 11). This can be attributed to relatively low mass content of Au in the BTO-Au nanocomposite. However, the increase of intensity of background with wavenumber in Raman spectrum in Figure 10 indicates the photo-luminescence (PL) of the BTO-Au sample at high Au loading. Observation of PL was also reported for BTO-Au nanocomposite prepared by sol-gel method [15]. In our conditions the PL of pure BTO NPs is wide and is red-shifted for more than 100 nm compared to BTO synthesized by chemical synthesis. Large width of PL spectrum is due to transitions between numerous levels of oxygen vacancies that are formed during laser ablation in H_2_O (see Figure 4, curve 2). Peak of PL observed in our work at 540 nm is attributed to the plasmonic luminescence of Au NPs supported on BTO NPs [30]. This peak was not reported for BTO-Au nanocomposite synthesized by sol-gel procedure [15].

Nanocomposites BTO-Au generated by both approaches contain Titanium oxides. This may be attributed to higher temperature of particles inside the laser beam due to the presence of Au NPs in them. Au enhances coupling of laser radiation to composite BTO-Au NPs. This consideration is partially supported by the fact that pure NPs of BTO are free of Titanium oxides. BTO is synthesized by reaction of BaCO_3_ and TiO_2_ at 1200 °C. One may suggest that enhanced absorption of BTO-Au NPs compared to pure BTO may results in higher temperature gained during the laser pulse, and the reverse reaction may occur. On the other hand, the presence of Ti oxides may result from the inhomogeneity of the pristine pellet, since Raman analysis does not confirm their presence. In part, this is confirmed by the Raman spectra of nanocomposites that do not show the presence of Titanium oxides. At this point the exact explanation of presence of Titanium oxides cannot be clarified.

## 5. Conclusions

Thus, laser synthesis of nanocomposites BaTiO_3_-Au has been successfully realized. Resulting nanocomposites are Au nanoparticles supported on crystalline nanoparticles of BaTiO_3_ with tetragonal lattice. Two approaches to the synthesis have been tested, either laser ablation of a bulk Au target in aqueous colloidal solution of BaTiO_3_ nanoparticles or laser irradiation of the mixture of Au and BaTiO_3_ colloids. Relatively long laser pulses are preferable for synthesis of both BTO and BTO-Au NPs, since the shape of BTO NPs by ablation with nanosecond pulses provides spherical NPs in contrast to short and ultra-short laser pulses. BTO NPs generated by laser ablation in H_2_O have absorption in the visible range of spectrum due to loss of oxygen. Au content in the nanocomposites BaTiO_3_-Au can be adjusted by either the time of laser ablation of a gold target or by relative fraction of colloidal solutions in the mixture. Synthesized nanocomposites have permanent and thermally stable absorption in the visible range of spectrum due to plasmon resonance of supported Au nanoparticles near 540 nm. Au content can be varied by adjusting the time of laser ablation of Au target in the colloidal solution of BTO NPs. Tests on the photocatalytic activity of the synthesized nanocomposites in light-induced water splitting are ongoing.

## Figures and Tables

**Figure 12:**
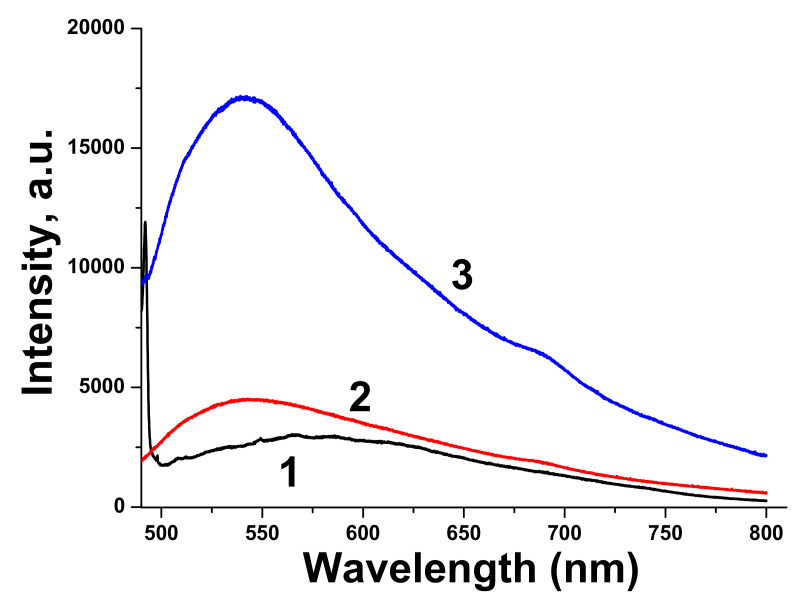
Luminescence spectra of BTO NPs ablated in H_2_O (1), Au-BTO NPs generated by laser ablation of Au target in aqueous solution of BTO NPs for 10 min (2), Au-BTO NPs generated by laser ablation of Au target in aqueous solution of BTO NPs for 15 min (corresponds to Figure 9) (3). Excitation wavelength of 473 nm.
